# PREREGISTRATION TO FACILITATE A REPLICABLE SCIENCE OF AGING

**DOI:** 10.1093/geroni/igz038.1481

**Published:** 2019-11-08

**Authors:** Walter R Boot

**Affiliations:** Florida State University, Tallahassee, Florida, United States

## Abstract

Psychology and other sciences have been in the midst of a replication crisis. One proposal to address this crisis is the preregistration of studies, including study hypotheses, methods, measures, and analysis approaches to reduce false positive findings resulting from “experimenter degrees of freedom.” This talk will explore the benefits, and also the challenges, of preregistration, along with common misconceptions about preregistration. A preregistration case study will be presented involving a series of experiments exploring different hypotheses regarding the mechanism behind changes in attentional processing associated with aging (http://doi.org/10.1525/collabra.26). This talk will present a brief tutorial of how to preregister studies and where to preregister them. The importance of preregistration for intervention studies will be emphasized.

